# Functional thresholds alter the relationship of plant resistance and recovery to drought

**DOI:** 10.1002/ecy.3907

**Published:** 2023-01-03

**Authors:** Johannes Ingrisch, Nikolaus Umlauf, Michael Bahn

**Affiliations:** ^1^ Department of Ecology University of Innsbruck Innsbruck Austria; ^2^ Department of Statistics University of Innsbruck Innsbruck Austria

**Keywords:** drought intensity, plant productivity, recovery, resilience, resistance, thresholds

## Abstract

The ecological consequences of future droughts are difficult to predict due to a limited understanding of the nonlinear responses of plants to increasing drought intensity, which can change abruptly when critical thresholds of drought intensity are crossed. Drought responses are composed of resistance and postdrought recovery. Although it is well established that higher drought intensity increases the impact and, thus, reduces plant resistance, less is known about how drought intensity affects recovery and how resistance and recovery are related. In this study, we tested the hypothesis that resistance, recovery, and their relationship change abruptly upon crossing critical thresholds of drought intensity. We exposed mesocosms of two monospecific stands of the common grassland species *Dactylis glomerata* and *Plantago lanceolata* to a large gradient of drought intensity and quantified the resistance and recovery of multiple measures of plant productivity, including gross‐primary productivity, vegetative height, Normalized Difference Vegetation Index, and aboveground biomass production. Drought intensity had nonlinear and contrasting effects on plant productivity during drought and recovery, which differed between the two species. Increasing drought intensity decreased the resistance of plant productivity and caused rapid compensatory growth during postdrought recovery, the degree of which was highly dependent on drought intensity. Across multiple response parameters two thresholds of drought intensity emerged, upon which we observed abrupt changes in plant resistance and recovery, as well as their relationship. We conclude that across gradients of drought intensity resistance and recovery are tightly coupled and that both the magnitude and the direction of drought effects on resistance and recovery can change abruptly upon specific thresholds of stress intensity. These findings highlight the urgent need to account for nonlinear responses of resistance and recovery to drought intensity as critical drivers of productivity in a changing climate.

## INTRODUCTION

Drought is considered the most widespread climate extreme affecting ecosystem productivity and the terrestrial carbon cycle (Reichstein et al., 2013; Sippel et al., 2018). Future drought regimes are likely to intensify with the projected increase in climate variability (Büntgen et al., 2021; Satoh et al., 2022; Spinoni et al., 2018), but the ecological implications remain uncertain due to a lack of understanding of how drought intensity drives ecological responses (Felton et al., 2021; Vicente‐Serrano et al., 2020). Ecological responses to a pulsed disturbance, such as drought, can be described by a system's resistance, that is, the ability of an ecosystem to withstand drought, and its recovery after disturbance (Ingrisch & Bahn, 2018). The latter ultimately defines whether a drought's impact can be overcome and, thus, whether drought effects are transient or persistent (Hillebrand & Kunze, 2020; Jentsch & White, 2019). Therefore, an understanding of the characteristics of an ecological response to disturbance requires a joint consideration of resistance and recovery (Hodgson et al., 2015; Ingrisch & Bahn, 2018; Nimmo et al., 2015). It has often been observed that resistance and recovery have a negative relationship, that is, that plants or ecosystems that possess a high resistance to drought recover slowly, and vice versa (de Boeck et al., 2018; Grime et al., 2000; Ingrisch et al., 2018). However, this does not consider the influence of the intensity of a drought event, which is a direct driver of vegetation responses to drought. Although resistance and recovery and their relationship determine the response trajectory of vegetation to drought events, we lack an understanding of whether and how they are affected by drought intensity.

**FIGURE 1:**
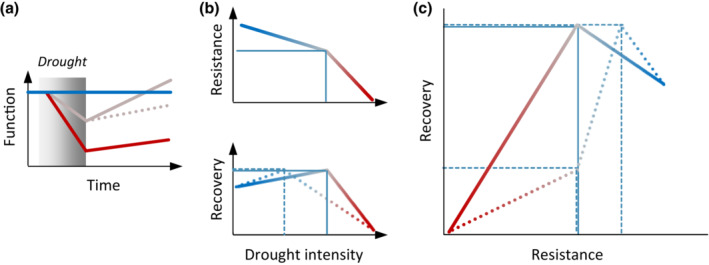
Illustration of ecosystem responses to pulsed stress disturbance, such as drought. (a) Temporal response of ecosystem function during stress (gray shading) and subsequent recovery. Different lines illustrate responses to stress of different intensities, where blue corresponds to an undisturbed system, gray to intermediate stress intensity, and red to severe stress. (b) Hypothesized response of resistance and recovery to stress intensity and (c) resulting relationship of resistance and recovery along a gradient of stress intensity. It is hypothesized that thresholds of stress intensity (vertical lines in [b]) cause abrupt changes in resistance and recovery and their relationship (c). Solid and dashed lines indicate two different scenarios, where the thresholds for resistance and recovery responses are similar (solid) or different (dashed), respectively. The color gradient of the lines depicts resistance and recovery illustrates stress intensity.

Stress responses are often nonlinear and can involve thresholds, that is, intensities of the stressor upon which the response changes abruptly (Groffman et al., 2006; Hillebrand et al., 2020). Our understanding of the occurrence and consequences of thresholds is limited (Berdugo et al., 2020; Dudney & Suding, 2020; Turner et al., 2020), partly because thresholds are difficult to detect empirically (Hillebrand et al., 2020). In response to drought, the resistance of plant productivity will generally decline with increasing stress intensity, and the decline can accelerate when critical thresholds (Figure 1b), such as hydraulic failure, turgor loss, and leaf senescence, are crossed (Munson et al., 2020; Verslues et al., 2006; Yan et al., 2017). Drought effects on plant recovery are less clear (Gilbert & Medina, 2016; Griffin‐Nolan et al., 2018; Müller & Bahn, 2022) and can be both negative and positive. Some studies have reported enhanced growth after drought related to increased nutrient availability (Hofer et al., 2017; Roy et al., 2016; Stampfli et al., 2018), whereas others have reported impaired postdrought productivity (Figure 1b) caused by stress‐induced damage, for example, impaired meristematic tissue and plant mortality (Reichmann et al., 2013). It has been suggested that the direction of drought effects on recovery is related to the degree of stress intensity and resistance (Griffin‐Nolan et al., 2018), and the change in direction can be abrupt, for example, when a plant's ability to avoid or tolerate stress is exceeded (Volaire, 2018). However, we lack experimental and observational evidence on how stress intensity alters the relationship of resistance and recovery. Hypothetically, the direction of the relationship between resistance and recovery can change upon a critical threshold (Figure 1c), as an increasing loss of resistance will no longer be causing an increase in recovery capacity but a loss. In consequence, passing such a threshold could disproportionately alter the response trajectory of the system and accordingly amplify drought impacts, for example, by resulting in prolonged negative drought effects instead of rapid compensatory growth.

Here we aimed to experimentally test the role of drought intensity on the resistance and recovery of plant productivity on two common grassland species. We hypothesize that resistance, recovery, and their relationship change abruptly at critical thresholds of drought intensity. Specifically, we expected that (i) a decline in the resistance of plant productivity would accelerate when a threshold of drought intensity was reached (Figure 1b), (ii) plant regrowth would increase with increasing intensity of the antecedent drought (“compensatory growth”) but slow down above a threshold of drought intensity (drought‐induced damage to plant tissues) (Figure 1b), and (iii) that these differential responses would cause a nonlinear relationship between resistance and recovery (Figure 1c). We studied the drought response of two grassland species, the grass *Dactylis glomerata* and the forb *Plantago lanceolata*. The two species are common, coexist in European grassland systems, and are representative of functional groups of grasses and forbs, which have been shown to differ vastly in their responses to drought (Hoover et al., 2014; Mackie et al., 2019; Wilcox et al., 2020). We grew the species in monospecific mesocosms in a common‐garden experiment and experimentally imposed a pulse drought event. To detect potential nonlinear responses to drought stress intensity, we used a gradient design (Kreyling et al., 2018) covering a broad range of drought intensities. This intensity gradient was implemented by excluding precipitation and adding defined amounts of water to obtain a broad range of soil water deficits (SWDs). We quantified drought effects on multiple measures of plant productivity during the period of drought, over the course of subsequent weeks of recovery, and in the year following the drought. We assessed the effects of drought intensity and thresholds on resistance–recovery relations by combining a bivariate framework with threshold regression analysis.

## METHODS

### Study design

We studied the drought responses of two different grassland species, *D. glomerata* (*var. Tandem*) and *P. lanceolata* grown in monocultures in a common‐garden experiment located at the Botanical Garden in Innsbruck [605 m above sea level (asl), 47.267628 N, 11.380426 E]. *D. glomerata* (grass) and *P. lanceolata* (forb) are perennial C3 species common in European grasslands. Seeds of both plant species were sourced from the Climgrass Project at HBLFA Raumberg‐Gumpenstein, Austria (Piepho et al., 2017), and were germinated in a greenhouse in April 2019. Four weeks after sowing, mesocosms (diameter 20 cm, height 25 cm) were planted with 10 seedlings of the respective plant species in a standardized, predefined grid. Mortality was checked weekly in the first month after planting, and a total of two dead individuals were replaced. Mesocosms were filled with a dry equivalent of 5000 g of homogenized and sieved (5 mm) field soil. Each mesocosm consisted of two pots with identical diameters; the inner one held the soil and plants and was stacked into the outer one, which was installed in a sand bed to buffer diel fluctuations in soil temperature. The inner pot had holes at the bottom to allow draining of soil water. Mesocosms were arranged randomly in the common garden under a rain‐out shelter, which was covered with transparent ultraviolet (UV)‐permissive foil (Lumisol clear AF, Folictec, Westerburg, Germany, light transmittance >90%) during the drought treatment.

### Experimental drought treatment

The drought treatment imposed a gradient of drought intensity following a gradient design (Kreyling et al., 2018). For each species, we created 14 levels of drought intensity, whereby the well‐watered end of the gradient (80% of field capacity) served as undisturbed baseline and was replicated four times, and each drought level was assigned a predefined SWD (Appendix S1: Table S1). The drought treatment was imposed by covering the mesocosms with a rain‐out shelter, with each mesocosm being allowed to dry down to its predefined target SWD and maintained at that level by regular addition of the required amount of water (Puértolas et al., 2017; Turner, 2019). Throughout the study, soil water content (SWC) and SWD are expressed relative to the SWC at field capacity. Mesocosm soil water status was determined gravimetrically based on pot weight (*m*_is_), soil dry mass (*m*_soil_), and water content at field capacity ($m_{H_{2}O,\mathrm{fc}}$)

(1) $$\mathrm{SWD}=\left(1-\frac{m_{\text{is}}-m_{\text{soil}}}{m_{H_{2}O,\mathrm{fc}}}\right)\times 100\%.$$

Water content at field capacity of each mesocosm was determined before the drought experiment started and 2 days after a large precipitation event, when pots were assumed to be at their respective field capacity (Sinclair et al., 2017; Turner, 2019). Pots were weighed and soil dry weight—calculated from soil bulk density and soil volume—was subtracted. Soil bulk density was determined based on two additional, unplanted, but otherwise identical mesocosms, by measuring soil volume and soil dry weight. We considered the effect of biomass growth on our pot weights negligible, given that the maximum error on the SWD at peak drought introduced by plant growth was in the range of 0.7%–2.8%, which corresponds to 0.1% (dry) to maximum 1% (well‐watered) of field capacity.

The imposed soil water gradient ranged from a SWD of 20% of field capacity (well‐watered baseline) to 95% of field capacity (Appendix S1: Table S1). Mesocosms were randomly assigned a target SWD. When the drought treatment started, mesocosms were weighed every 1–2 days. Once the pots reached their predefined SWD, they were watered to maintain this water status (Appendix S1: Figure S1). The drought treatment lasted for 23 days (Appendix S1: Table S2) and was terminated by watering all pots to field capacity. Afterwards, pots were maintained at well‐watered conditions for the recovery period.

### Measurements

Throughout the period of the experiment, we repeatedly assessed gross primary productivity (GPP), Normalized Difference Vegetation Index (NDVI), and vegetative height as nondestructive measures of plant productivity.

GPP, that is, the CO_2_ uptake rate of the plant canopy, has been shown to be a suitable measurement to compare the drought responses of grassland communities (Ingrisch et al., 2018) and was measured using ecosystem chambers. In this way, net ecosystem exchange (NEE) and ecosystem respiration (*R*_eco_) were measured pairwise with closed dynamic chambers, as described by Ingrisch et al. (2018). Briefly, for the NEE measurement a cylindrical transparent chamber (diameter 25 cm, height 50 cm) equipped with a CO_2_ sensor (GMP343, Vaisala, Helsinki, Finland) and a water vapor sensor (HMP 75, Vaisala, Helsinki, Finland), and a fan was placed airtight on the mesocosms. CO_2_ concentration in the chamber was measured for 1 min in 5‐s intervals. Then *R*_eco_ was measured using a dark chamber. Measurements were done in the late morning on days with a mostly clear sky, and photosynthetic active radiation (PAR) was recorded with each measurement (PQS 1 PAR Quantum Sensor, Kipp & Zonen, Delft, the Netherlands). Fluxes were calculated from the slope of a linear regression between time and chamber concentration and quality controlled based on visual inspection of data and the quality of the linear fit (Pirk et al., 2016). GPP was calculated as the difference between the corresponding NEE and *R*_eco_ measurements. Throughout this paper, GPP is assigned a positive sign.

The NDVI is a spectral indicator that correlates with the structural and functional properties of the canopy and has been recommended as a nondestructive measure of stress responses (Rossi et al., 2019). It was measured on the mesocosm level using spectral reflectance sensors (SRS NDVI, Metergroup, Munich, Germany) attached to a custom‐made mobile logging unit. The unit consisted of a sensor head holding an upward‐facing hemispherical sensor and a directed downward‐facing sensor. The sensor head was mounted to an extension arm (length 1.5 m) held by a tripod and aligned by a bubble level. For measurements, pots were lifted out of the ground and placed under the sensor head to achieve maximum comparability between measurements. The sensor head was centered 50 cm above the pot rims, with the tripod positioned to eliminate light interference. This height allowed the mesocosms to be exactly in the sensor field of view. To prevent reflections from disturbing the measurement, the surroundings of the measured pots were covered with a black cloth at the height of the pot rims. Each pot was measured three times and averaged prior to further analysis. Measurements were taken regularly (4× drought, 7× recovery) between 10 and 12 h on days with predominantly clear skies. The NDVI was calculated from the respective wavelength signals of the up‐ and downward‐facing sensors (Gamon et al., 2015).

Vegetative height was used to assess biomass production over time (Lavorel et al., 2011). The average and maximum vegetative height of each mesocosm was determined between twice a week and fortnightly using a ruler. Aboveground biomass was sampled destructively (i) 1 week prior to the drought treatment, (ii) at peak drought, (iii) in the late growing season 2.5 months after the drought, and (iv) at peak biomass in the subsequent year (Appendix S1: Table S2). During harvest, plants were cut approximately 3 cm above soil level and dried at 80°C prior to weighing. Leaf nitrogen concentration (LNC) in biomass at the first recovery harvest was determined by cutting the aboveground biomass into 2‐ to 3‐cm pieces, mixing, and taking a subsample, which was ball‐milled into a fine powder and weighed into a tin capsule. Nitrogen content was measured with a Flash EA1112 elemental analyzer (Thermo Fisher Scientific). The total amount of nitrogen was calculated based on N concentration and biomass dry weight. Relative leaf‐water content (rLWC) was determined at peak drought on the youngest adult leaf of three plant individuals per mesocosm. Leaves were cut, immediately weighed for fresh weight (*m*_fresh_), and rehydrated following protocol (Pérez‐Harguindeguy et al., 2016) by wrapping them into moist paper towels for 24 h and storing them at 4°C. Following measurement of the leaf saturation weight (*m*_sat_), samples were dried at 80°C for 72 h prior to measurement of the dry weight (*m*_dry_). rLWC was calculated as rLWC (%) = (*m*_fresh_ − m_dry_)/(*m*_sat_ − *m*_dry_) × 100. Microclimatic parameters (photosynthetically active radiation, air temperature, and relative humidity) were measured continuously (S‐THB‐M002 and S‐LIA‐M003, Onset Computer Corporation, Bourne, MA, USA) inside the rain‐out shelter at 1.5 m above ground (Appendix S1: Figure S7).

### Data analysis

We quantified resistance and recovery based on the plant responses over the course of the experiment. Resistance was determined based on measurements conducted at peak drought, when the maximum concurrent impact of the drought treatment occurred. Recovery is a dynamic process that we analyzed through repeated measurements in the weeks following the drought. We conducted in‐depth analysis of drought intensity effects on recovery (threshold detection and resistance–recovery relationships, see following discussion) of the different response parameters for specific measurement days. Where repeated measurements were available (NDVI, GPP, vegetative height) we selected measurement days that showed the strongest effect of SWD on the respective parameter, since drought effects generally diminished toward the end of the growing season.

**FIGURE 2:**
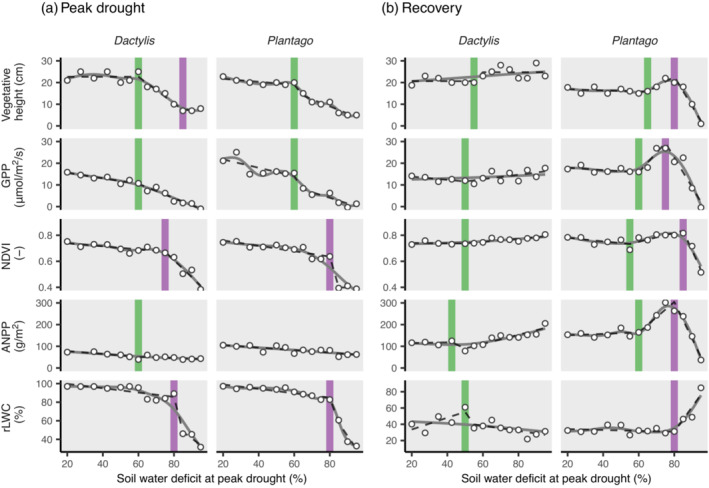
Drought responses and response thresholds. Effects of drought intensity at end of drought treatment, expressed as soil water deficit at peak drought (percentage of field capacity), on plant performance (a) at peak drought and (b) during selected days of recovery period. Points represent actual measurements, gray curves show generalized additive models fitted to the data, dashed lines denote best‐fit threshold regression models (Appendix S1: Table S3), with corresponding threshold intensity shown as thick vertical lines and color distinguishing between lower (green) and upper (purple) threshold (Figure 4). Peak drought measurements were taken during the last days of the drought treatment, the days of recovery measurement for the different variables are given in Appendix S1: Table S3 and indicated in Figure 3. ANPP, aboveground net primary productivity; GPP, gross primary productivity; NDVI, Normalized Difference Vegetation Index; rLWC, relative leaf water content.

Thresholds of SWD for drought resistance and recovery were identified using threshold regression models (Berdugo et al., 2020; Fong et al., 2017). The analysis is based on the procedure described by Berdugo et al. (2020) and was performed for the different plant responses (Figure 2) on a daily basis. First, we evaluated whether responses to drought intensity were nonlinear. For this, responses of each plant species were modeled with linear and nonlinear [quadratic and generalized additive models (GAMs)] regression, with the target SWD as explanatory variable. The Akaike information criterion (AIC) was used to decide whether nonlinear models provided a better fit than linear models in each case. Nonlinearity is a prerequisite for the existence of thresholds, and therefore threshold regression models were applied to nonlinear relations only (chngpt R package; Fong et al., 2017). Following Berdugo et al. (2020) and Groffman et al. (2006), we consider two types of thresholds: Continuous thresholds change the relationship (slope) of the response variable and the stressor (SWD), whereas discontinuous thresholds are values of the stressor, which change the value of the response abruptly, that is, change the intercept at a given point. Continuous thresholds were modeled by segmented regressions, allowing for either one or two thresholds—discontinuous thresholds by step regression (change in the intercept of the model at threshold) or a combination of step and segmented (segmented) regressions (change in intercept and slope of the model at threshold). Each of these models was fit to the responses previously characterized as nonlinear. The best model for each response was selected primarily based on AIC, though the following additional filter criteria were applied: Segmented regressions were only considered when each segment of the regression consisted of a minimum of three observations, and positive intercept changes in step and segmented regressions during drought were omitted. Threshold detection was repeated with 1000 bootstrapped samples to estimate the distribution of each threshold.

To visualize the temporal dynamics of plant productivity in response to the drought treatment, we modeled a response surface of plant productivity (GPP, NDVI, vegetative height) over SWD and time using GAM (Hastie & Tibshirani, 1990). These models were specified with

(2) $$\begin{array}{c} Y=\beta_{0}\;+\;\beta_{1}\cdot {\text{Spec}}_{\mathrm{Pla}}\;+\;f_{1}\left(Doy\right)\;+\;f_{2}\left(\mathrm{SWD}\right)\\ +\;f_{3}\left(Doy,\mathrm{SWD}\right)\;+\;f_{4}\left(Doy\right)\cdot {\text{Spec}}_{\mathrm{Pla}}\\ +\;f_{5}\left(\mathrm{SWD}\right)\cdot {\text{Spec}}_{\mathrm{Pla}}\;+\;f_{6}\left(Doy,\mathrm{SWD}\right)\cdot {\text{Spec}}_{\mathrm{Pla}}\\ +\;f_{7}\left(\mathrm{pot}\right)+\varepsilon , \end{array}$$

with $\varepsilon ∼N\left(0,\sigma^{2}\right).$ Here Doy denotes day of the year and Y represents the response variable (GPP, NDVI, and vegetative height). In this model, the coefficient $\beta_{0}$ is the intercept, and $\beta_{1}$ reflects the effect of plant species *Plantago* (for estimating species‐specific effects, *Dactylis* was chosen as the reference category in the regression models). The functions $f_{1},\ldots ,f_{3}$ of covariates Doy and SWD have possible nonlinear effects and are modeled nonparametrically using regression splines. The model term *f*_3_ accounts for possible interactions of Doy and SWD on the response of variable *Y*. Similarly, functions $f_{4},\ldots ,f_{6}$ represent possible nonlinear deviations for the species *Plantago* (so‐called effect modifiers; Fahrmeir et al., 2013). The function *f*_7_ is a pot‐specific random effect since data are nested in pots across time. This effect is vanishingly small owing to the study design and does not affect the results. All models were calculated using the mgcv R package (Wood, 2011). All model terms were tested for significance and showed extremely low *p*‐values (Wood, 2013). Model residuals were visually checked for normal distribution (Appendix S1: Figure S8), and the models did not exhibit autocorrelation in the residuals (Appendix S1: Figure S9). The drought phase (Doy 183–212) and the recovery phase (Doy 213–287) were modeled separately. The recovery phase is defined as the period between the end of the drought to the final seasonal harvest in late fall.

Finally, resistance and recovery of the different response variables were mapped into a bivariate resistance–recovery framework (Bahn & Ingrisch, 2018; Ingrisch & Bahn, 2018). This was based on the responses of the different variables, predicted by GAMs during peak drought and during the selected days of recovery (Figure 1) and aboveground biomass in the subsequent year. The predicted values of the response Y along the SWD axis were normalized to the corresponding baseline value $Y_{\text{base}}$ to calculate the normalized resistance and recovery. For each SWD the corresponding normalized resistance and recovery were mapped to the bivariate scheme. Furthermore, thresholds identified in drought and recovery responses were added. All data analysis was done in R version 4.03 (R Core Team, 2020).

## RESULTS

### Drought

The dry‐down of mesocosms during the drought treatment was fast but slowed down with increasing SWD. All mesocosms reached their target SWD within the period of the drought treatment, and a high level of agreement between target SWD and actual SWD was achieved (Appendix S1: Figure S1).

Plant productivity at the end of the drought treatment, measured by vegetative height, NDVI, GPP, and aboveground net primary productivity (ANPP), generally declined with increasing drought intensity (Figure 2a, Appendix S1: Figures S2 and S3). For all productivity measures except ANPP of *Plantago*, this decline was best described by nonlinear relationships, as indicated by the model AIC of linear versus nonlinear models (Appendix S1: Table S3). The leaves of both species maintained a high rLWC up to a SWD of 80% of field capacity (FC), upon which they dried out quickly to less than 40% (Figure 2a).

We identified threshold responses in the resistance of several response parameters in both plant species (Figures 2 and 4). A first threshold occurred at a SWD of 50%–60% of FC (shown in green in Figures 2 and 4), at which point the decline of vegetative height and GPP accelerated. A second threshold at ca. 80% of SWD was associated with a rapid decline of NDVI and rLWC.

### Recovery

**FIGURE 3:**
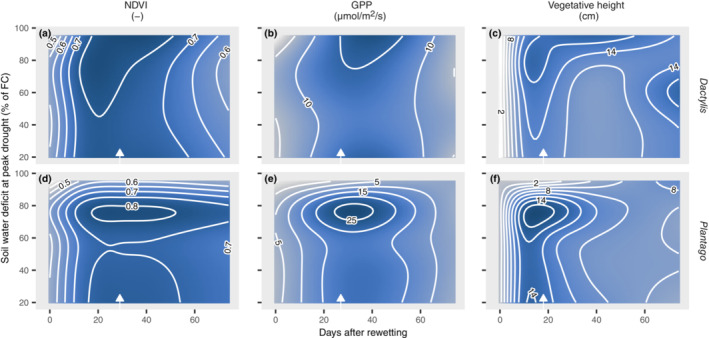
Response surface of different plant productivity measures (a, b) Normalized Difference Vegetation Index (NDVI), (c, d) gross‐primary productivity (GPP) and (e, f) vegetative height during recovery period. The *x*‐axis indicates days after the combined rewetting and harvest event, the *y*‐axis shows drought intensity at the peak of the drought (at end of drought treatment), expressed as soil water deficit (percentage of field capacity). The response surface is based on repeated measures and modeled with generalized additive models (see *Methods*, Appendix S1: Tables S4 and S5). Color intensity and isopleths denote value of productivity measures. White arrows at bottom of each panel indicate dates selected for threshold analysis (Figures 2 and 4).

**FIGURE 4:**
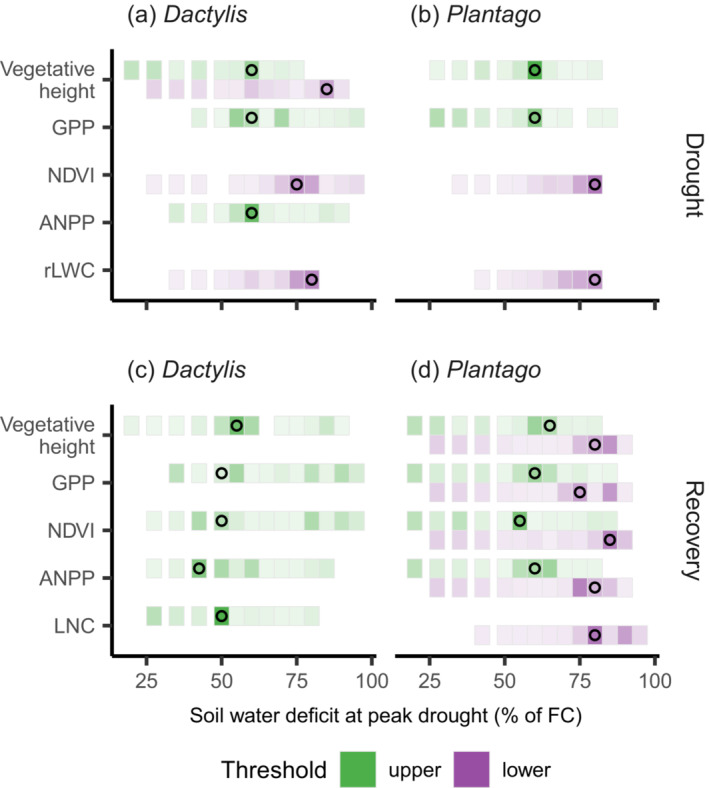
Distribution of response threshold estimate for different response variables during (a, b) peak drought and (c, d) recovery. Color density shows distribution of bootstrapped threshold estimates. Colors denote a lower and upper threshold. Points denote threshold of best model fit shown in Figure 2. ANPP, aboveground net primary production; FC, field capacity; GPP, gross primary productivity; NDVI, Normalized Difference Vegetation Index; rLWC, relative leaf water content.

Plant regrowth after the combined rewetting and harvest at peak drought was generally fast. All plant individuals survived in all mesocosms except for the highest drought treatments of *Plantago*, where the survival rate was 80% and 20% for SWD of 90% and 95%, respectively. Repeated nondestructive measurements of the different productivity measures showed an immediate onset of regrowth, and within 20–30 days after rewetting NDVI, GPP, and vegetative height reached their maximum (Figure 3, Appendix S1: Figure S2). SWD at peak drought had a significant effect on the recovery dynamics (Appendix S1: Table S5) and affected the rate and degree of regrowth. Generally, in the recovery period, mesocosms recovering from drought were more productive than the corresponding baseline controls, and this feature was consistent for both species and across the different productivity parameters studied. However, postdrought recovery dynamics differed between the two species. Recovery of *Dactylis* increased with increasing SWD along the entire gradient of drought intensity (Figures 2b and 3). In contrast, the highest recovery of *Plantago* was reached by mesocosms that were exposed to a drought intensity of ca. 80% of FC, whereas above that SWD regrowth was slowed distinctly (Figures 2b and 4). This might be partly related to the die‐off of some individuals in the two highest drought intensities, but importantly, surviving individuals clearly showed slower growth rates. The degree of the observed postdrought overcompensation was higher for *Plantago* than for *Dactylis*. At 2.5 months after the drought, the ANPP of the drought‐affected *Dactylis* and *Plantago* overshot baseline mesocosms by up to 75% and 100%, respectively. Ten months after the drought, the ANPP of *Dactylis* showed the negative effects of drought intensity, whereas *Plantago* showed positive drought legacies on ANPP (Appendix S1: Figure S5). Nitrogen pools and concentrations in aboveground biomass 2.5 months after the drought were strongly increased in *Plantago*'s recovery from the drought but not in *Dactylis* (Figure 2b, Appendix S1: Figure S4).

Threshold regression models were calculated for all measured productivity parameters on selected days of the recovery period, where the drought effects on recovery were most pronounced (Figure 3, Appendix S1: Table S3). The level of drought intensity that triggered postdrought overcompensation (Figure 2b) was clearly indicated across all productivity parameters by a threshold at ca. 50% and 60% of SWD for *Dactylis* and *Plantago*, respectively (Figure 4). A second threshold was identified for *Plantago* at a SWD of ca. 80% of FC, at which point productivity rapidly declined.

### Bivariate resistance–recovery analysis

**FIGURE 5:**
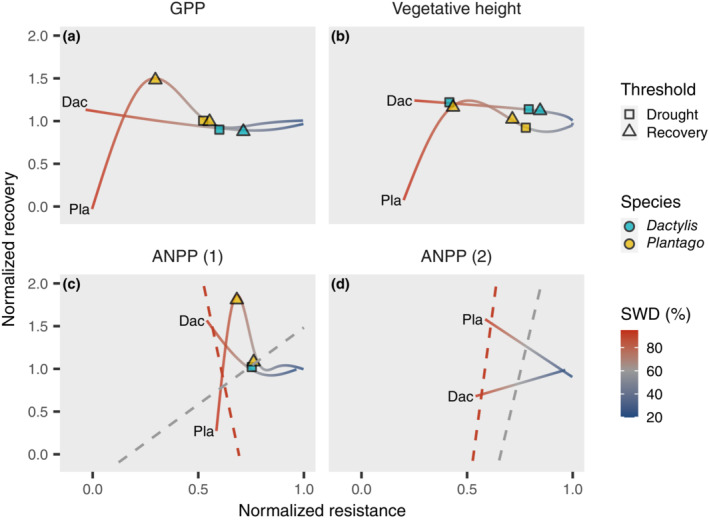
Relationships of resistance and recovery for different measures of productivity. Bivariate analysis of drought resistance at peak drought and recovery for (a) gross primary productivity (GPP), (b) vegetative height, (c) aboveground net primary productivity (ANPP) 2.5 months after drought, and (d) in subsequent year. Both resistance and recovery are normalized to the baseline (well‐watered mesocosms). Resistance and recovery are predicted from generalized additive models (Figure 3). Line colors indicate drought intensity, expressed as soil water deficit (SWD) at peak drought (percentage of field capacity). The points denote the thresholds derived from the response curves; point shape indicates whether the threshold value was derived from the drought or the recovery response. Point colors distinguish the plant species *Dactylis glomerata* (Dac) and *Plantago lanceolata* (Pla). The two dashed lines in the ANPP panels (c) and (d) indicate isopleths of SWDs across the two species. The isopleths indicate the interspecific relationship of resistance and recovery at similar stress intensities, where a negative slope corresponds to a trade‐off between resistance and recovery and a positive slope implies the opposite.

We mapped the response curves of resistance and recovery to SWD derived for the different productivity parameters into a bivariate framework of resistance and recovery (Figure 5). The analysis showed that recovery was largely affected by drought resistance and that this dependency was highly nonlinear and changed when the thresholds of drought resistance and recovery were crossed. Within each species the relationship between resistance and recovery for the different response parameters was generally similar in the first year (Figure 5a–c) and only differed in the magnitude of resistance and recovery responses. However, the relationship of resistance and recovery differed between the two species. For *Dactylis*, recovery generally increased with a loss of resistance, whereas *Plantago* showed an optimum curve, with the highest recovery at an intermediate resistance of the respective parameter and a loss of recovery at low resistance. Ten months after the drought had ended, ANPP(2), the relationship between impact and recovery was positive for *Plantago* and negative for *Dactylis*.

## DISCUSSION

The primary aim of this study was to investigate how drought intensity affected the resistance and recovery of plant productivity and whether critical thresholds affected their relationship. We found nonlinear relationships between plant resistance and recovery across the imposed gradient of stress intensity (Figure 2). These responses evolved around two thresholds of stress intensity that affected various plant response parameters during drought and recovery (Figure 4). Mapping resistance and recovery in a comparative resistance–recovery framework (Bahn & Ingrisch, 2018; Hodgson et al., 2015; Ingrisch & Bahn, 2018) (Figure 5) showed a highly nonlinear relationship that changed when thresholds of drought intensity were passed, which resulted from the different effects of stress intensity on resistance and recovery.

### Thresholds in plant resistance and recovery

Drought intensity had negative nonlinear effects on plant productivity, with a distinct SWD threshold at ca. 60% of field capacity (Figures 2a and 4, Appendix S1: Figure S2). This threshold marks an acceleration of drought effects on vegetative height and GPP, while leaf hydration was largely maintained also beyond that threshold. This threshold likely reflects the onset of dehydration avoidance mechanisms related to moderate drought (Volaire, 2018; Zwicke et al., 2015), which have been associated with a pronounced reduction of stomatal conductance at the cost of productivity, maximization of water uptake, and protection of cells from low water potential (Verslues et al., 2006; Yan et al., 2017). Interestingly, the same threshold of drought stress marks the onset of positive effects on plant regrowth after drought, which led to a pronounced overcompensation of vegetative height, GPP, NDVI, and aboveground biomass during the recovery phase (Figures 2b and 3). Interestingly, both the rate and the magnitude of plant regrowth were strongly related to drought intensity and were generally more pronounced for *Plantago* than for *Dactylis*. Enhanced regrowth of drought‐stressed plants could result from rapid use of carbohydrates stored below ground, including osmoprotective compounds accumulated during drought (Hasibeder et al., 2015; Ingrisch et al., 2020; Thomas, 1991), as well as enhanced postdrought N availability (Hofer et al., 2017; Roy et al., 2016). The latter was supported by our finding that the aboveground tissue N concentration and N pool were increased in *Plantago* after 2.5 months of recovery (Figure 2b, Appendix S1: Figure S3). This is consistent with previous observations that shoot N concentration is increased after drought (Ingrisch et al., 2018; Mackie et al., 2019; Roy et al., 2016), possibly due to an enhanced microbial N turnover (Schimel, 2018) and remobilization of N stored in roots (Heckathorn & DeLucia, 1994). However, for *Dactylis*, whose drought recovery was less pronounced than for *Plantago*, we did not find increased tissue N concentrations or pools during recovery (Figure 2b, Appendix S1: Figure S4). Since the soil substrate was identical for both species, we conclude that the differences in postdrought N dynamics were species‐specific and driven by differences in physiology and/or the associated microbial communities. These results suggest that the physiological plant responses of dehydration avoidance do not only enhance the immediate chances of drought survival (Volaire, 2018; Zwicke et al., 2015) but also have positive effects on recovery capacity.

A second response threshold (Figures 2 and 4; SWD of 70%–80% of field capacity) marked the onset of severe drought stress and was related to leaf dehydration (decline in rLWC) and senescence, a halt of photosynthetic activity (GPP, Figure 2a) and a collapse of the canopy (reflected in strongly reduced vegetation height and NDVI, Figure 2a). The massive decline in plant functioning during severe drought was associated with reduced recovery rates of *Plantago* but not *Dactylis* (Figure 2b, Appendix S1: Figure S6). Notably, the poor recovery of *Plantago* at the highest drought intensity was not primarily driven by limited plant survival but mostly by significantly slower regrowth rates of surviving individual plants. This suggests that the two species differ in their ability to protect from or tolerate dehydration in roots and leaf meristems, both important mechanisms for drought survival and recovery (Verslues et al., 2006; Volaire et al., 2020; Zwicke et al., 2015).

### Drought legacies in subsequent year

The legacies of drought for plant productivity prevailed also 1 year after the experiment. Interestingly, these legacies differed between species and contrasted the recovery patterns in the first year, in that *Plantago* showed a positive response to drought intensity, whereas *Dactylis* showed a negative response. This suggests that, on the one hand, negative drought effects on regrowth can be overcome, though with considerable lags; on the other hand, fast initial regrowth and rapid overcompensation do not preclude negative drought legacy effects on growth in the subsequent year. Such limited agreement between immediate regrowth and long‐term recovery has been suggested to result from a combination of drought tolerance mechanisms and resource acquisition strategies (Craine et al., 2013; Wilcox et al., 2021; Zwicke et al., 2015). Postdrought regrowth is directly related to adaptive mechanisms enhancing drought tolerance, like the storage and mobilization of carbohydrates (Ingrisch et al., 2020; Volaire et al., 2020; Zwicke et al., 2015). This could explain why drought intensity thresholds were also evident in the immediate plant regrowth. In contrast, productivity in the year following the drought was found to be affected by N availability (Griffin‐Nolan et al., 2019; Mackie et al., 2019), suggesting that *Plantago* or its associated microbial communities might have a higher efficiency for N remobilization or uptake from soil than *Dacytlis* (Henneron et al., 2020). These results support the notion that recovery can be achieved by different mechanisms that may differ across stress intensity, species (Craine et al., 2013; Pérez‐Ramos et al., 2013), and temporal scales (Müller & Bahn, 2022). They also indicate that drought recovery assessments obtained at a single point in time (e.g., Hoover et al., 2014; Isbell et al., 2015; Stuart‐Haëntjens et al., 2018) might provide an incomplete and potentially biased picture of drought recovery.

### Toward an understanding of resistance–recovery relations across species and scales

Our results highlight the role of drought intensity and response thresholds for plant resistance and recovery. This raises several questions for future research related to our understanding of drought responses and resistance–recovery relations (i) beyond individual species and (ii) at higher ecological scales.

First, given the pronounced interspecific differences, plant drought responses should be assessed across a broader range of species, which would make it possible to generalize the results beyond the species‐specific results presented here. It remains to be explored whether and to what degree traits and trait syndromes can serve to predict species' responses to drought across gradients of drought intensity. For example, resistance and recovery have been shown to be related to the fast–slow economic spectrum of plants (de Boeck et al., 2018; Grime et al., 2000; Ingrisch et al., 2018; Wilcox et al., 2021), where conservative species are characterized by higher resistance and acquisitive species by faster recovery. However, it remains unclear whether the fast–slow spectrum only affects the magnitude of the response or also the position of the response thresholds. Consequently, we lack an understanding of how drought intensity alters the general trade‐off between resistance and recovery. In our study, to compare resistance–recovery relations of the two plant species at similar drought intensities, the isopleths of SWD were mapped in the bivariate scheme for ANPP (Figure 5c,d). A negative slope of the isopleth corresponds to a trade‐off between resistance and recovery because the system with lower resistance has the higher recovery, whereas a positive slope implies the opposite. The analysis of ANPP at the first recovery sampling indicates that drought intensity can change the slope of these isopleths and that an inverse relationship between resistance and recovery did not occur at low stress intensities. This suggests that stress intensity modulates interspecific resistance–recovery relations. Future studies should further explore this interaction across a wide fast–slow spectrum of plant strategies.

Second, the pronounced effects of drought intensity on plant response trajectories raise questions about how these effects scale up to resistance and recovery responses at higher ecological scales. The differential drought sensitivity of species within communities presents an important mechanistic pathway buffering community‐ or ecosystem‐level responses by enhancing the asynchrony and reordering of species abundances (Craven et al., 2018; Felton & Smith, 2017; Hoover et al., 2014; Mariotte et al., 2013). Our results suggest that drought intensity might be critical in determining which species drive postdrought community reordering since the two species displayed different optima of stress intensity on regrowth rates. Thus, the postdrought trajectory of community composition might be critically dependent on drought intensity and change abruptly when thresholds of individual species are crossed. However, these considerations do not account for ecological processes operating at the community level, which can modulate the drought responses of individuals (Felton & Smith, 2017). Facilitative and competitive plant–plant interactions can modulate drought effects on individual plants, for example, by altering nutrient or water supply, and, furthermore, the direction of such plant–plant interactions can change with stress intensity and over the course of a drought (Jentsch et al., 2011; Ploughe et al., 2019). Such community‐level processes preclude any straightforward scaling from individual plant to community‐level responses (Felton & Smith, 2017; Wilcox et al., 2021) and highlight the need for future research to study drought intensity effects across multiple levels of organization in assembled or natural environments.

We conclude that plant drought resistance and recovery are tightly coupled but that thresholds in drought responses can alter both the degree and the direction of drought effects, especially as concerns the postdrought compensatory growth dynamics in both the same and the following year. Furthermore, the responses differed considerably between the two studied species in particular with regard to their ability to recover after a drought. Thus, the resistance–recovery relationship is critically dependent on drought intensity and the considered timescale of recovery and species identity. Our study highlights the importance of drought intensity and the critical role of functional thresholds for plant responses to drought and emphasizes the need to understand nonlinear threshold responses across a broad range of species and ecological scales in order to predict the consequences of a more extreme future climate.

## AUTHOR CONTRIBUTIONS

Johannes Ingrisch designed the experiment with input from Michael Bahn. Johannes Ingrisch conducted the experiment and analysis with the help of Nikolaus Umlauf and Michael Bahn. Johannes Ingrisch drafted the manuscript with the assistance of Michael Bahn. All authors contributed to the final draft.

## CONFLICT OF INTEREST

The authors declare no conflict of interest.

## Supporting information


Appendix S1.

## Data Availability

Data and code (Ingrisch, 2022) are available in Figshare at https://doi.org/10.6084/m9.figshare.19248266.
