# Supplementary material for: Functional thresholds alter the relationship of plant resistance and recovery to drought
Source: Ecology. 2023 Jan 3;104(2):e3907. doi: 10.1002/ecy.3907 (PMC10078541; doi:10.1002/ecy.3907)
Supplement: Supplementary file 1 — Appendix S1. [file ECY-104-0-s001.pdf]

Journal: Ecology

Supporting Information

Ingrisch, J., N. Umlauf, and M. Bahn.

Functional thresholds alter the relationship of plant resistance and recovery to drought.

Appendix S1

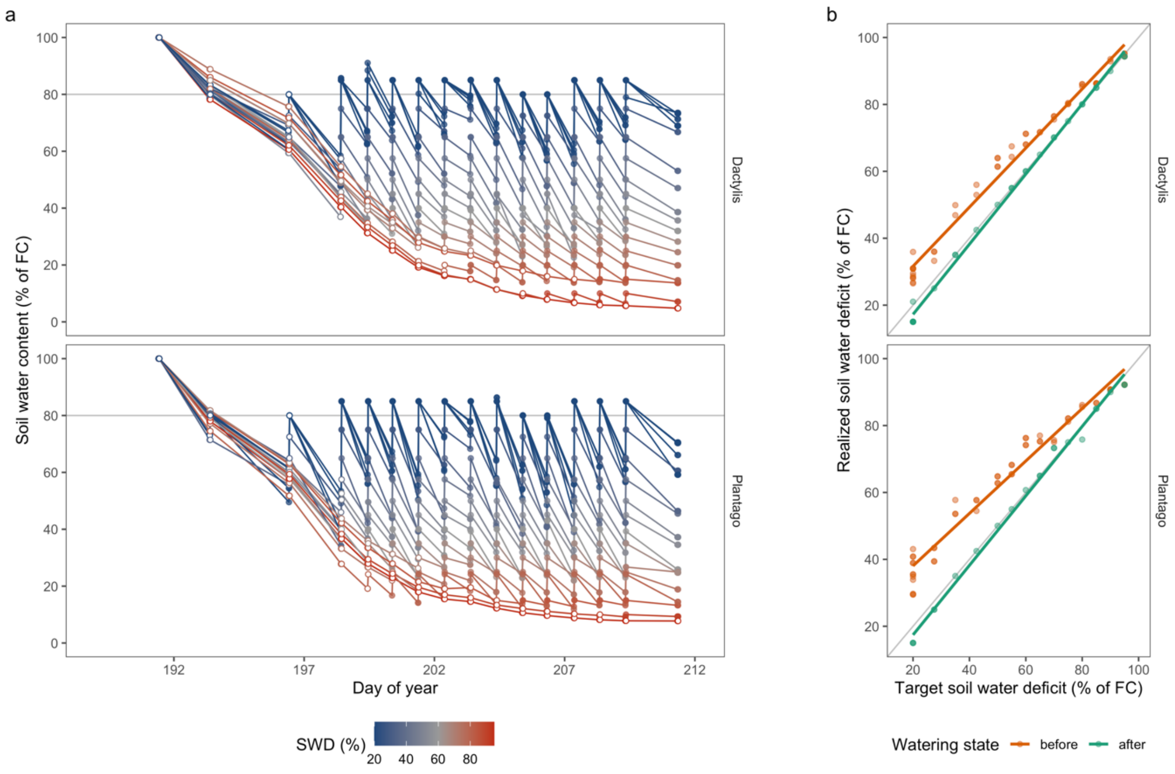

Figure S1: ***Soil water content during the drought treatment****. Soil water content of mesocosms in response to drought treatment. (a) Time course of individual mesocosms. Colors denote the target soil water deficit (SWD; % of field capacity). Vertical offsets in lines denote the increase of SWC by watering. Open points denote the dry-out phase, closed symbols denote that the mesocosm has reached its target SWD. Grey horizontal line indicates target SWC of baseline (80%). (b) Realized soil water deficit in relation to target soil water on the last two days of the drought treatment. Colors denote water content before and after watering, lines show the corresponding linear regression. The grey lines indicates the 1:1 relationship. Abbreviations: FC: field capacity.*

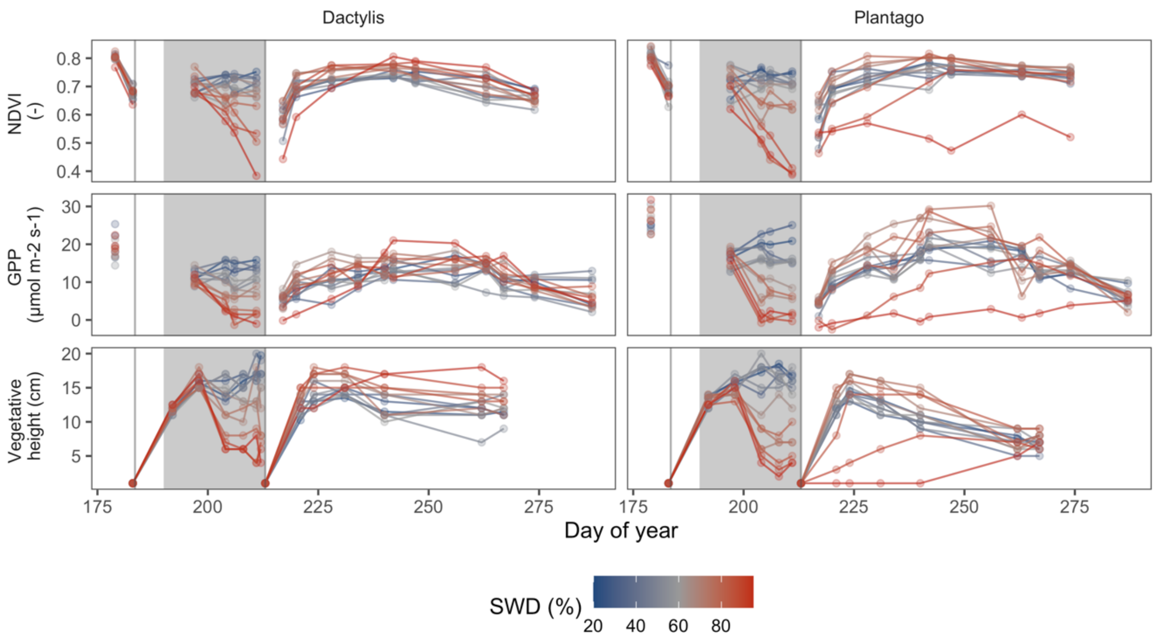

Figure S2: ***Seasonal time course of productivity measures*** *NDVI (Normalized Difference Vegetation Index), GPP (Gross Primary Productivity) and vegetative height. The period of the drought treatment is shaded grey, aboveground harvest before drought and at peak drought is indicated by vertical lines. Color of the lines and points indicates the drought intensity treatment expressed as soil water deficit (SWD in % of field capacity) imposed on mesocosms at peak drought. Each line represents an individual mesocosm, lines are broken upon harvest dates.*

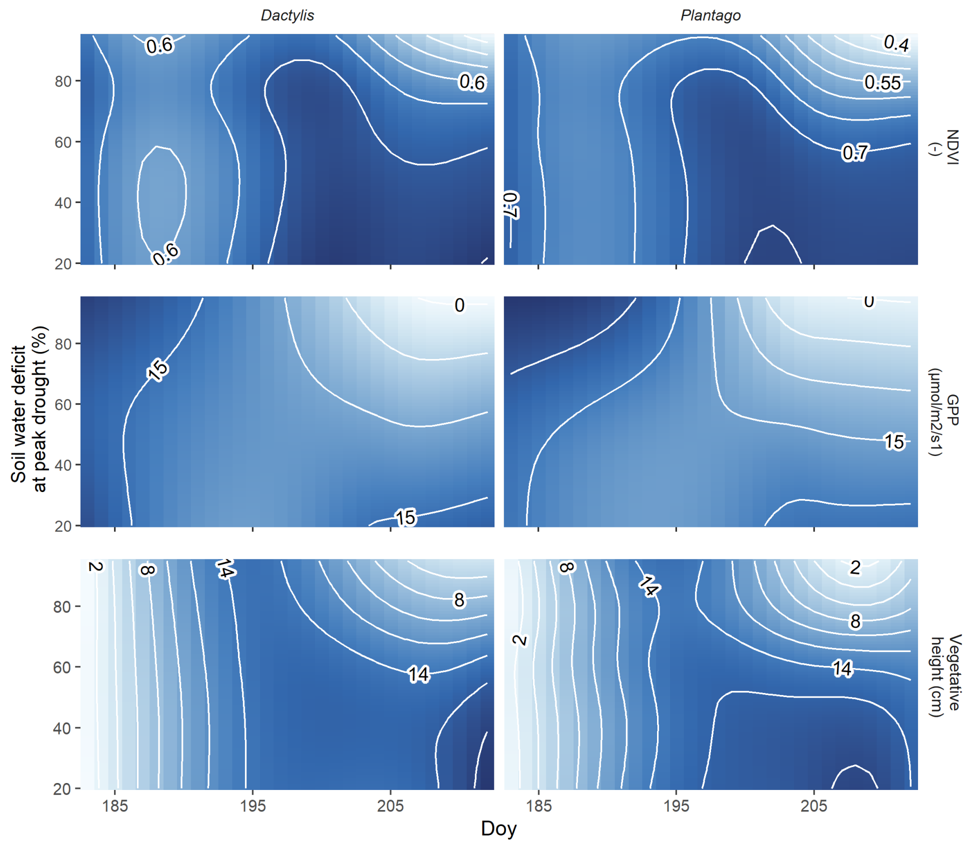

Figure S3: Response surface of different plant productivity measures normalized-difference vegetation index (NDVI), gross-primary productivity (GPP) and vegetative height during the drought treatment. The x-axis indicates day of the year, the y-axis shows drought intensity at the peak of the drought (at the end of the drought treatment), expressed as soil water deficit (% of field capacity). The response surface is based on repeated measures and modelled with GAMs (see methods, Tables S4, S5). Color intensity and isopleths denote the value of the productivity measures.

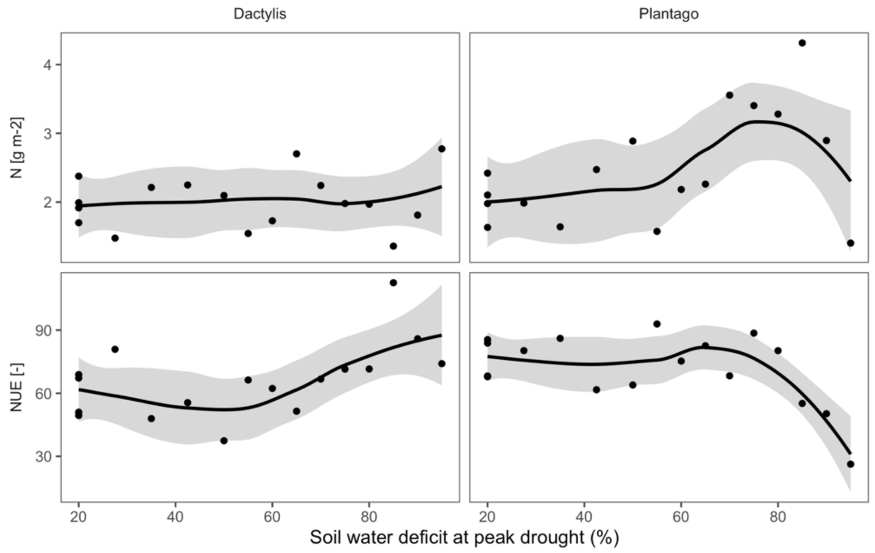

Figure S4: ***Shoot nitrogen during recovery.*** *Aboveground nitrogen pool and nitrogen use efficiency (NUE) in shoots sampled at the end of the recovery period. X-axis corresponds to the drought intensity at peak drought. NUE is calculated as the ratio of ANPP and the nitrogen pool. Lines indicate smoothing spline with confidence intervals to indicate overall trends.*

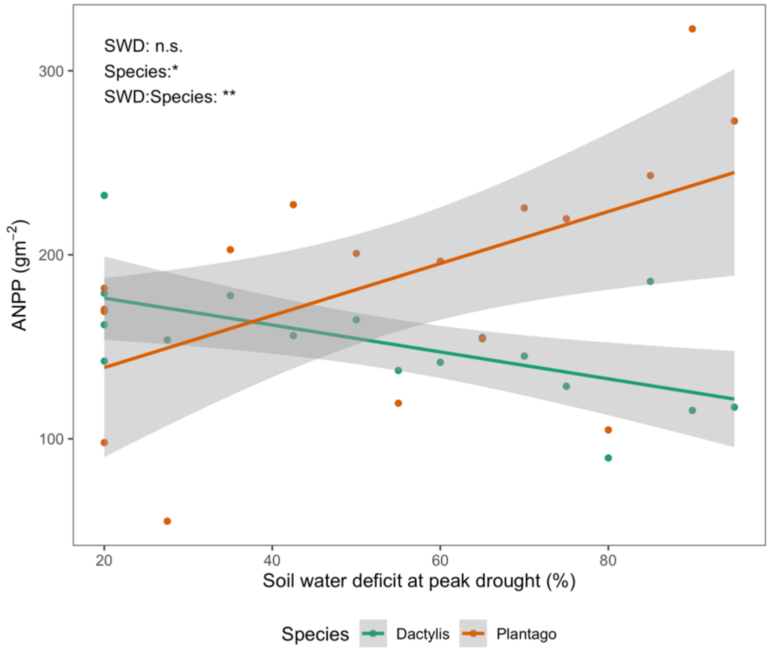

Figure S5: ***ANPP one year after the drought in relation to drought intensity at peak drought.*** *Points denote individual mesocosms, lines are linear regressions and confidence intervals. Statistics are derived from a linear model testing for SWD, Species and their interaction. Stars denote p-value of explanatory variable (*: $p<0.05$, **: $p<0.01$). Soil water deficit at peak drought refers to the drought intensity in the year previous year and is expressed in % of field capacity.*

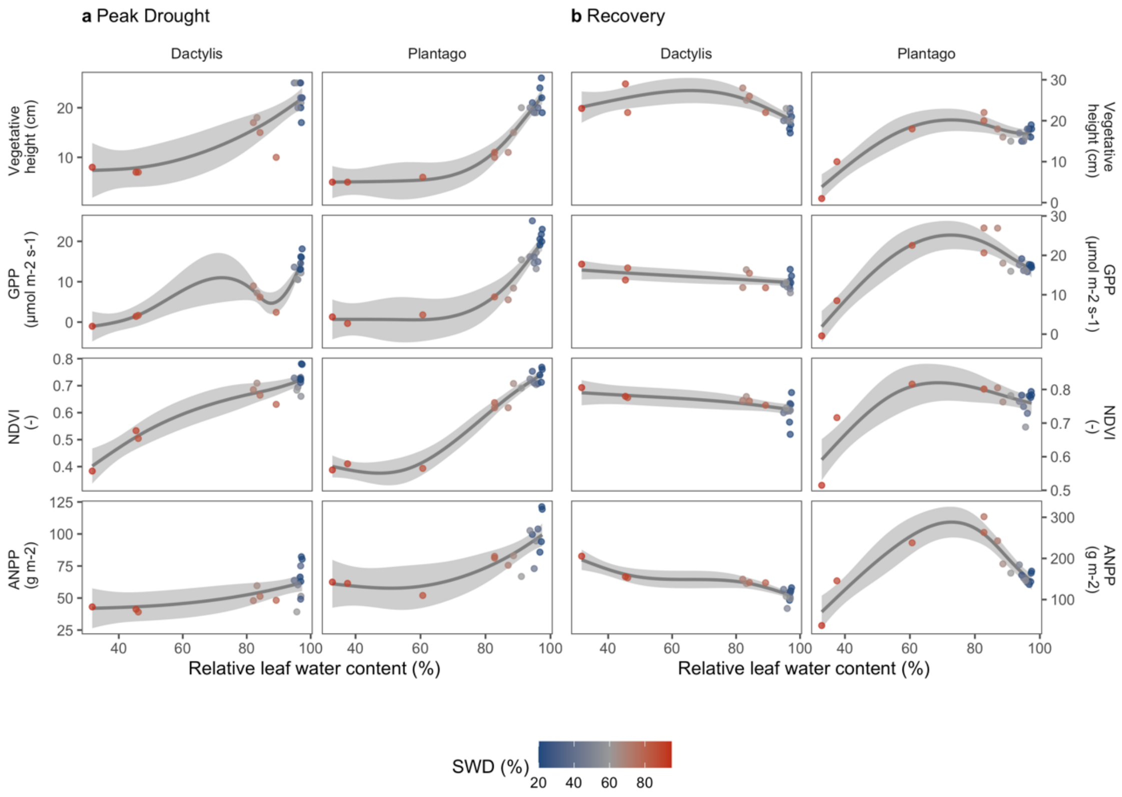

Figure S6: Productivity responses during (a) peak drought and (b) recovery in relation to the relative leaf water content measured in youngest adult leaves at peak drought. Colors denote drought intensity based on soil water deficit at peak drought (% of field capacity). Points denote individual mesocosms, lines are GAMs with confidence intervals. Recovery responses are measured on different days of the recovery period (see Fig 1, Fig S2 and Table S3).

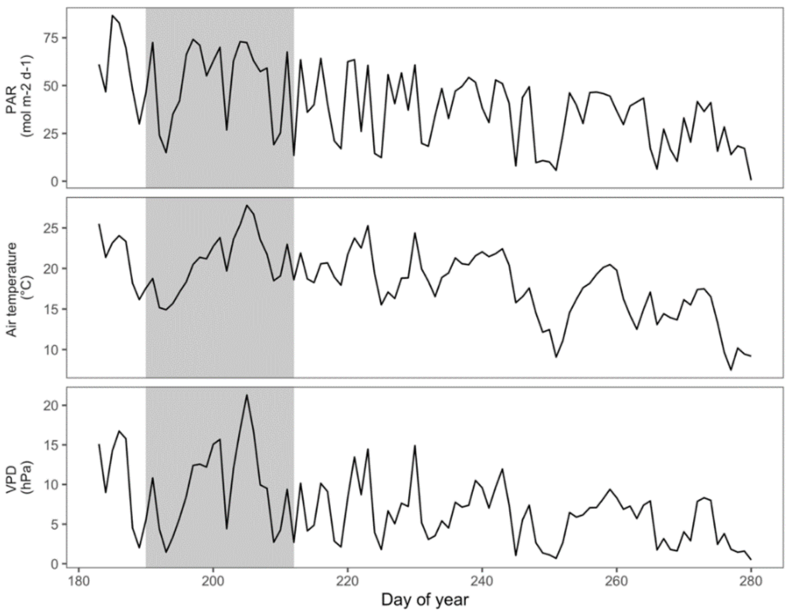

Figure S7: ***Microclimatic conditions in the common garden during the experiment.*** *Time course of daily sums of photosynthetically active radiation (PAR) and daily means of air temperature and vapor pressure deficit. The period of rain-out shelter closure is denoted by the grey shaded area.*

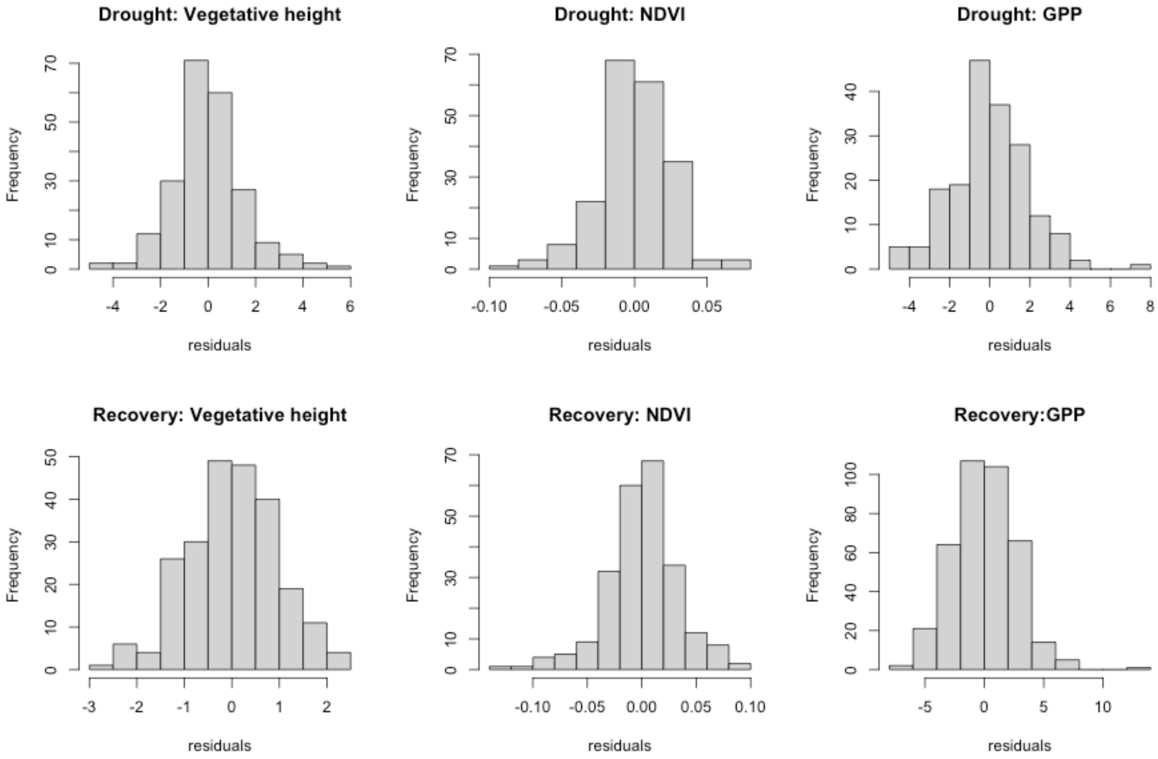

Figure S8: Distribution of residuals from the individual GAMs fitted for the three response variables for the drought (upper) and recovery (lower) period.

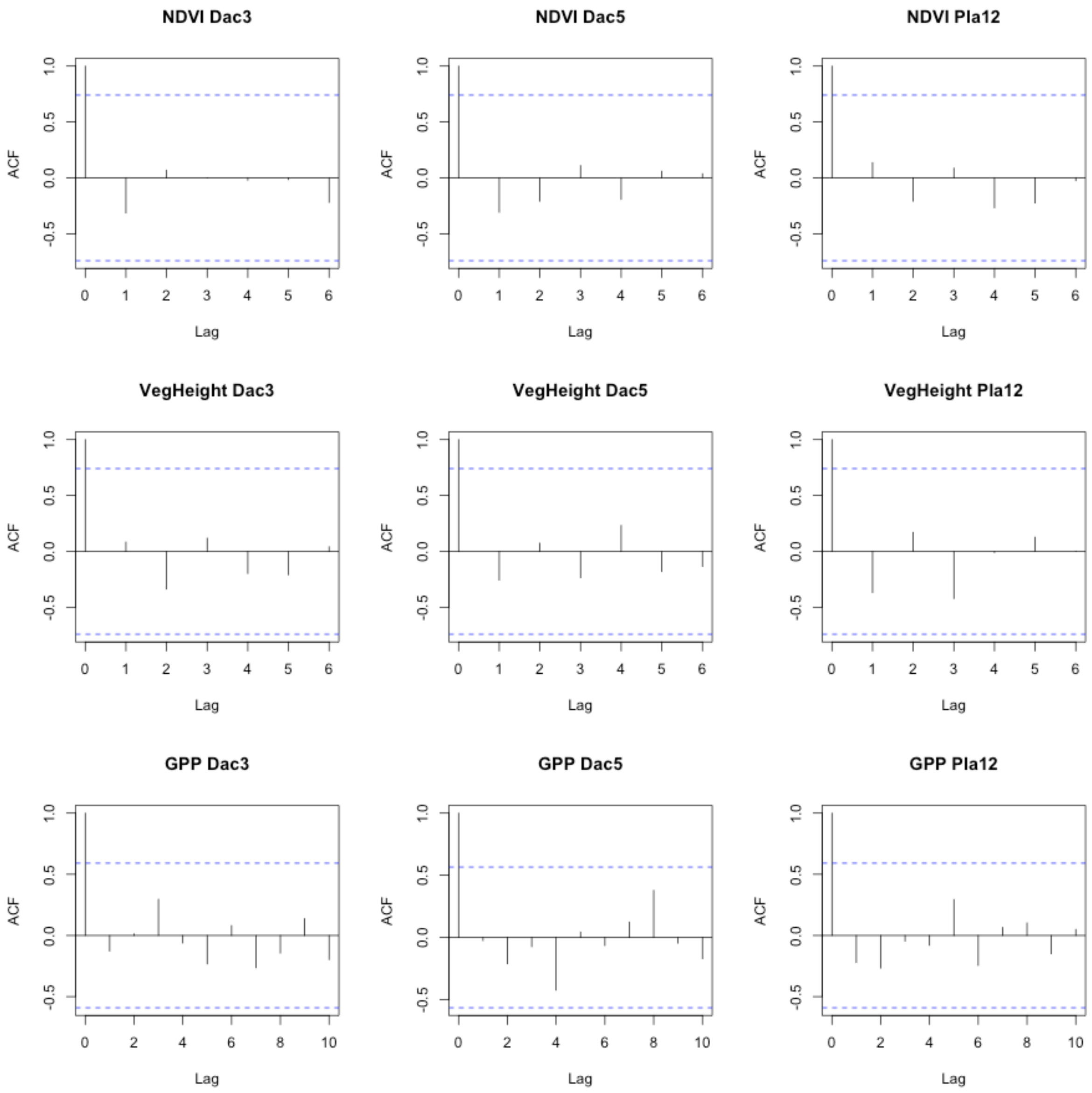

Figure S9: Empirical autocorrelation plots of the estimated residuals for a randomly selected subset of mesocosms. The plots indicate zero autocorrelation, because lag effects are insignificant.

Table S1: Target soil water content and corresponding soil water deficit of the different drought intensities imposed on the mesocosms. N denotes the number of replicates (n) within each treatment intensity and species set.

**Soil water content [% of field capacity]**	**Soil-water deficit (SWD) [% of field capacity]**	**N**
80	20	4
72.5	27.5	1
65	35	1
57.5	42.5	1
50	50	1
45	55	1
40	60	1
35	65	1
30	70	1
25	75	1
20	80	1
15	85	1
10	90	1
5	95	1

Table S2: Key dates of the experiment.

**Action**		**Date**
Planting	Seeding	9 Apr 2019
	Transplantation	9 May 2019
Harvest	Pre-drought	2 Jun 2019
	Peak drought	31 Jul 2019
	Recovery	15 Oct 2019
	2nd year	2 Jul 2020
Drought	Start	9 Jul 2019
	End	31 Jul 2019

Table S3: Model selection for threshold regression models. Akaike Information Criterion for the linear, non-linear and threshold model for each response variable and campaign as well as the selected model-type of the best fit model used to identify response thresholds. Doy gives the date of measurement used for the specific variable.

**Campaign**	**Variable**	**Plant**	**Doy**	**AIC**			**Best fit**
				Linear	nonlinear	Threshold model	Model type
Peak Drought	veg. height	Dac	212	79.74	70.41	62.63	M111
		Pla	211	68.49	61.16	50.37	Steg
	GPP	Dac	211	57.99	49.30	47.10	PW1
		Pla	211	67.13	68.39	65.94	Steg
	rLWC	Dac	212	118.38	105.27	86.72	Steg
		Pla	212	116.81	99.44	79.07	Steg
	NDVI	Dac	211	-33.80	-43.71	-55.28	PW1
		Pla	211	-28.92	-39.99	-58.54	Steg
	ANPP	Dac	212	89.43	90.51	89.11	PW1
		Pla	212	110.59	112.43	110.59	Lin
Recovery	veg. height	Dac	231	70.59	70.59	66.95	Step
		Pla	231	88.13	45.43	58.39	M111
	GPP	Dac	240	63.79	61.66	61.52	PW1
		Pla	240	98.31	73.83	73.87	M111
	LNC	Dac	288	105.03	102.67	97.95	Steg
		Pla	288	115.06	94.36	93.71	PW1
	NDVI	Dac	242	-74.63	-86.84	-77.49	PW1
		Pla	242	-28.39	-51.29	-56.26	M111
	ANPP	Dac	288	130.38	115.48	119.10	Steg
		Pla	288	161.43	121.66	131.19	M111

Abbreviations: Doy: day of year, Veg. height: vegetative height, GPP: gross-primary productivity, rLWC: relative leaf water content, NDVI: Normalized Difference Vegetation Index, ANPP: aboveground net primary productivity. AIC: Akaike Information criterion. Model types: Lin = linear regression, PW1 = segmented model with single threshold, M111 = segmented model with two threholds, Steg = Stegmented regression.

Table S4: Summary results of the generalized additive models describing the effects of drought intensity and day of the year on response variables during recovery (see Fig. 2).

Campaign	Variable	df	nobs	R.sq
Recovery	Veg. height	59.703	238.000	0.950
	NDVI	33.832	236.000	0.763
	GPP	51.236	384.000	0.760

Abbreviations: df: degrees of freedom, nobs: Number of observations used, R.sq: R squared statistic.

Table S5: Model information of the generalized additive model describing the effects of drought intensity and day of year on response variables during recovery (see Fig. 2)

Variable	Regression term	edf	ref.df	T-statistic	p
veg height	ti(Yday):PlantDac	3.97	4.00	468.45	0.000
	ti(Yday):PlantPla	3.98	4.00	365.14	0.000
	ti(SWD):PlantDac	1.81	1.93	16.14	0.000
	ti(SWD):PlantPla	3.94	3.96	29.58	0.000
	ti(Yday,SWD):PlantDac	11.81	14.08	4.07	0.000
	ti(Yday,SWD):PlantPla	14.44	15.64	16.69	0.000
	s(Plot)	17.76	30.00	1.80	0.000
NDVI	ti(Yday):PlantDac	3.89	3.99	56.38	0.000
	ti(Yday):PlantPla	3.85	3.99	67.83	0.000
	ti(SWD):PlantDac	2.68	2.98	1.19	0.346
	ti(SWD):PlantPla	3.95	3.98	30.59	0.000
	ti(Yday,SWD):PlantDac	6.83	8.95	3.57	0.000
	ti(Yday,SWD):PlantPla	1.00	1.00	0.05	0.818
	s(Plot)	9.63	30.00	0.51	0.035
GPP	ti(Yday):PlantDac	3.10	3.52	63.08	0.000
	ti(Yday):PlantPla	3.46	3.80	136.38	0.000
	ti(SWD):PlantDac	2.57	2.74	1.40	0.359
	ti(SWD):PlantPla	3.97	3.98	22.17	0.000
	ti(Yday,SWD):PlantDac	7.64	10.03	2.81	0.002
	ti(Yday,SWD):PlantPla	11.64	13.43	5.70	0.000
	s(Plot)	16.86	30.00	1.60	0.000

Abbreviations: edf: effective degrees of freedom, ref.df: reference degrees of freedom. Yday: Day of the year, SWD: soil water deficit at peak drought. PlantDac: Dactylis glomerata, PlantPla: Plantago lanceolata
